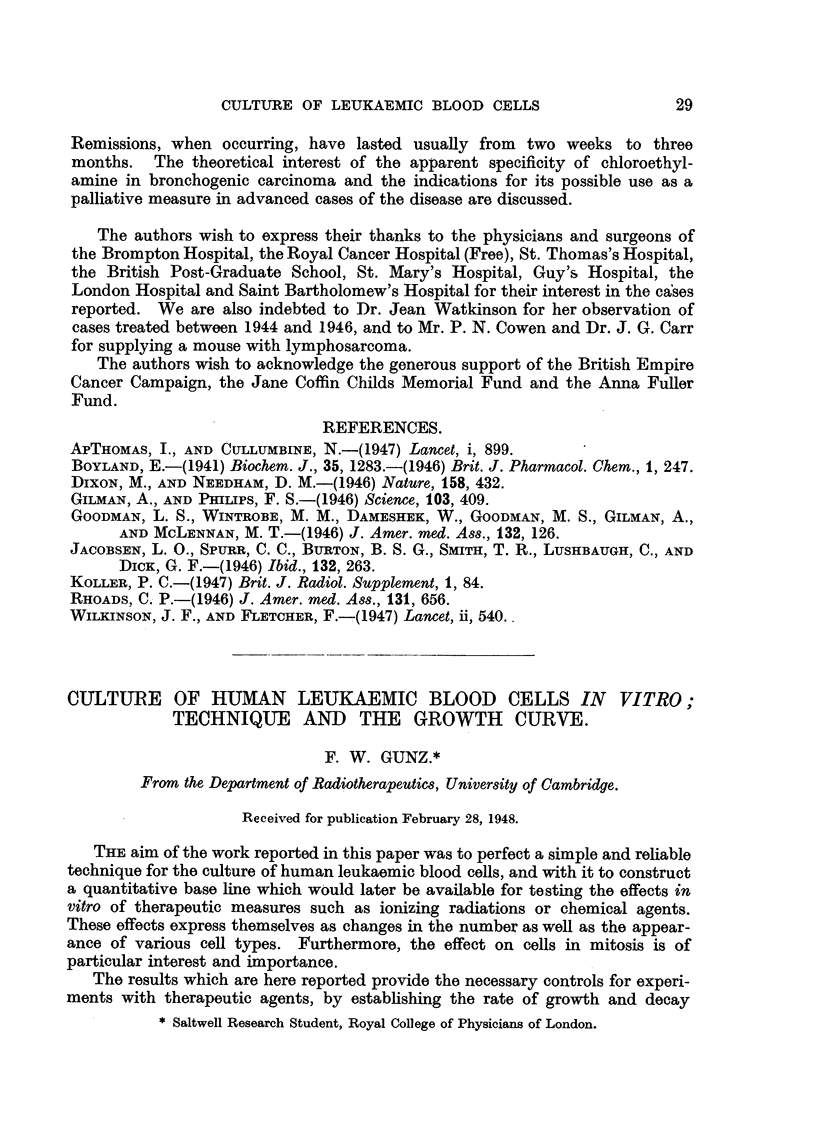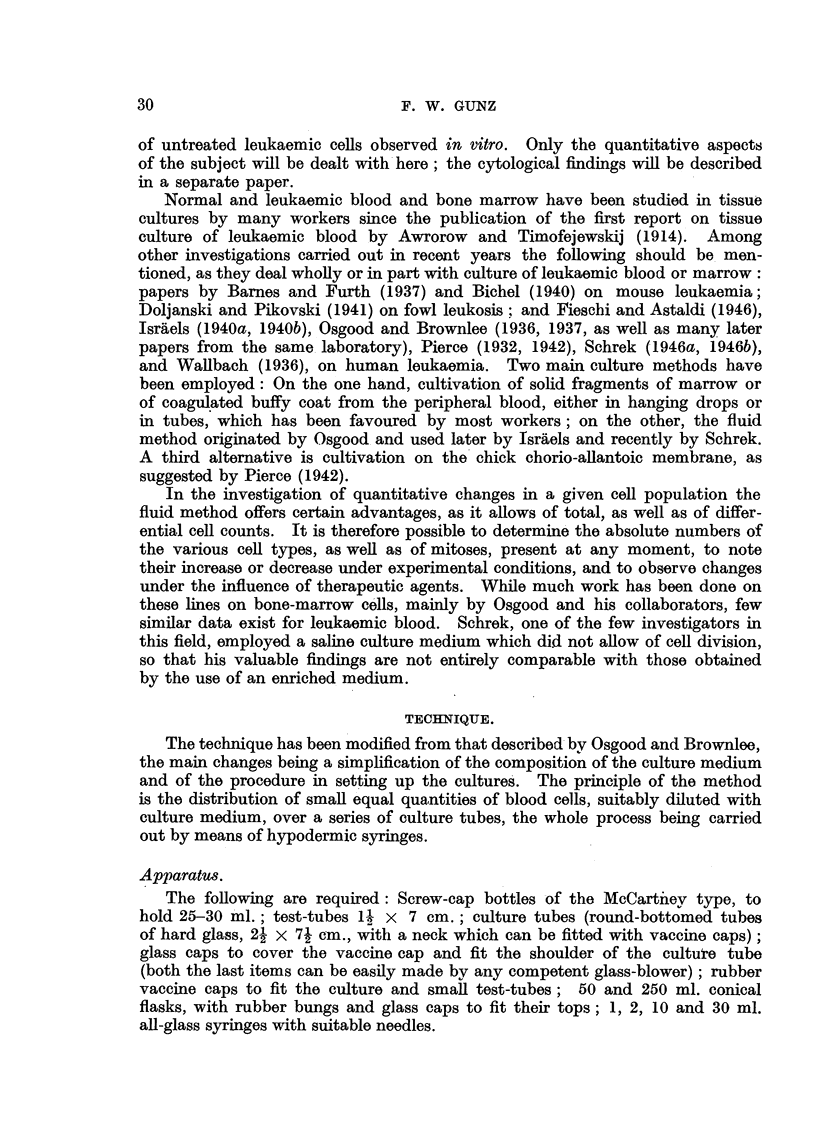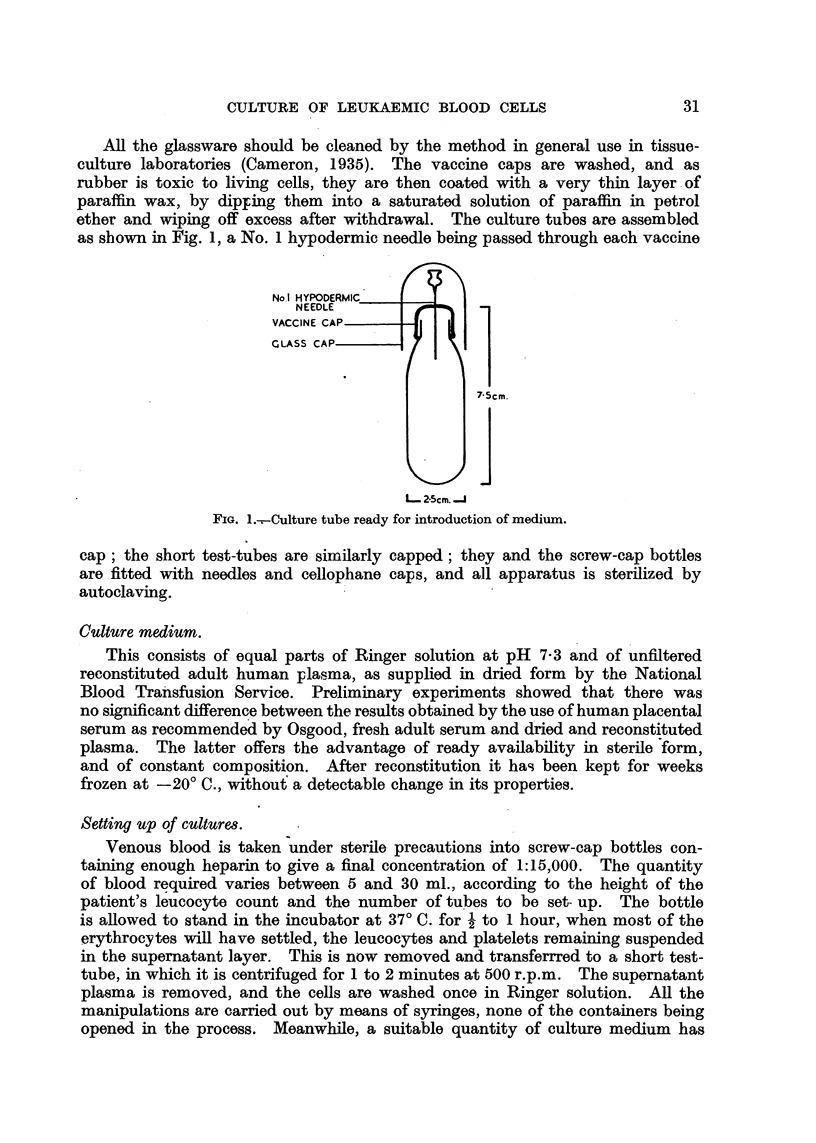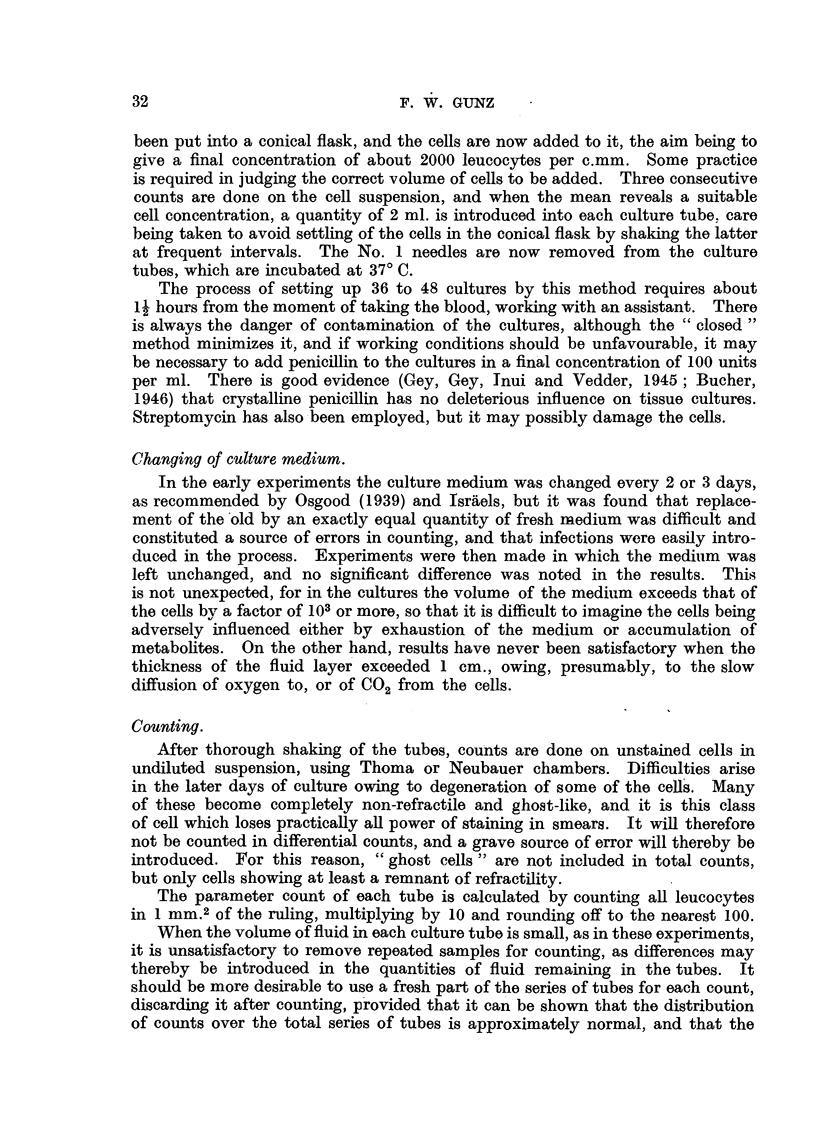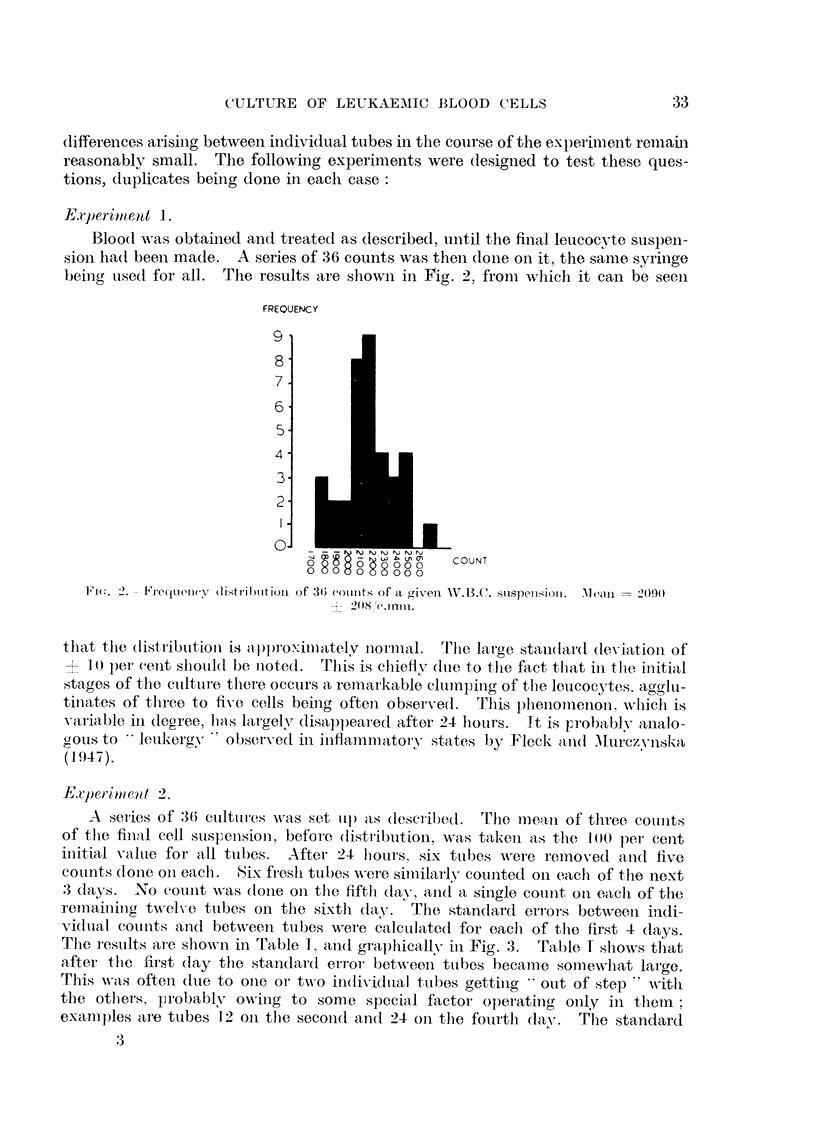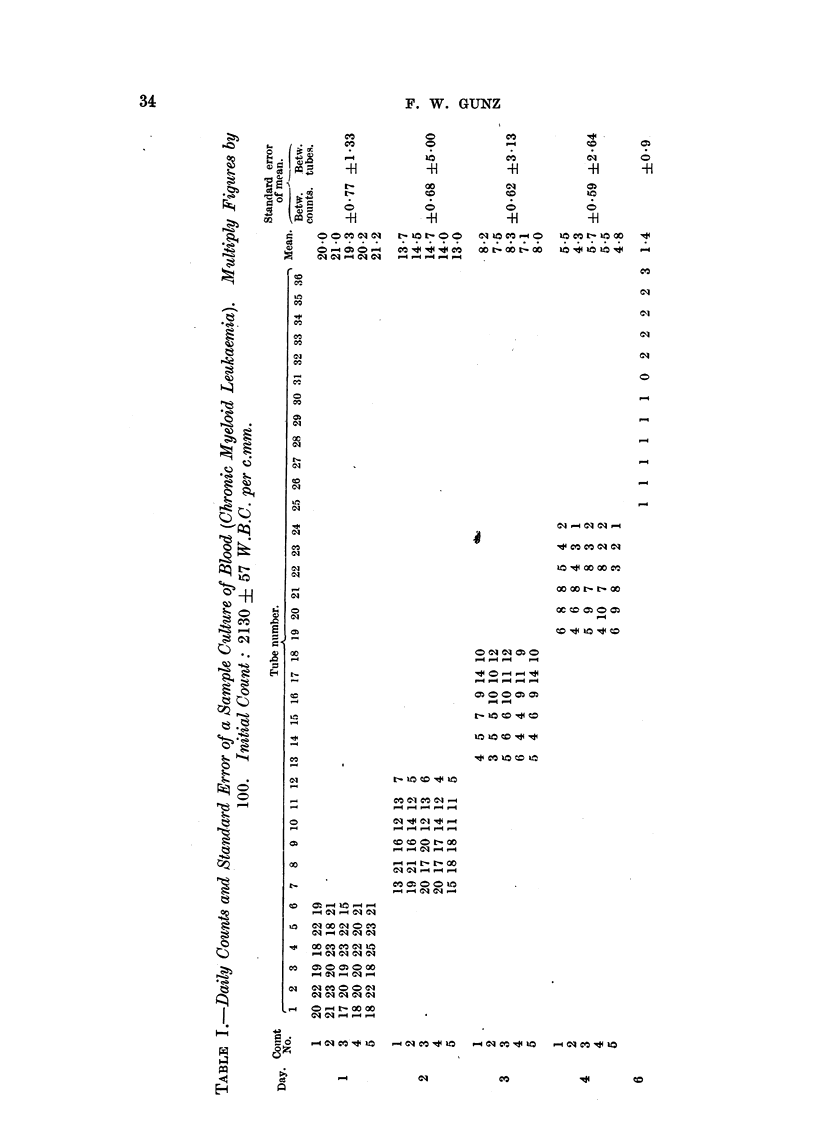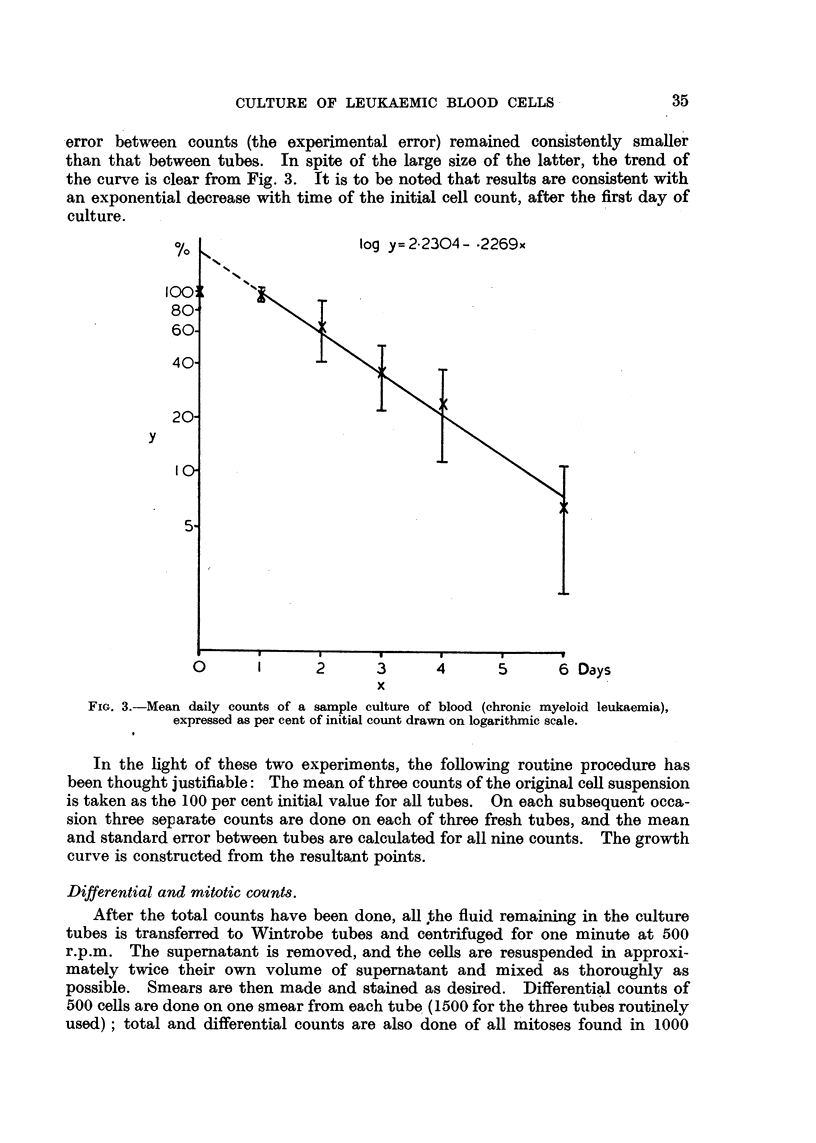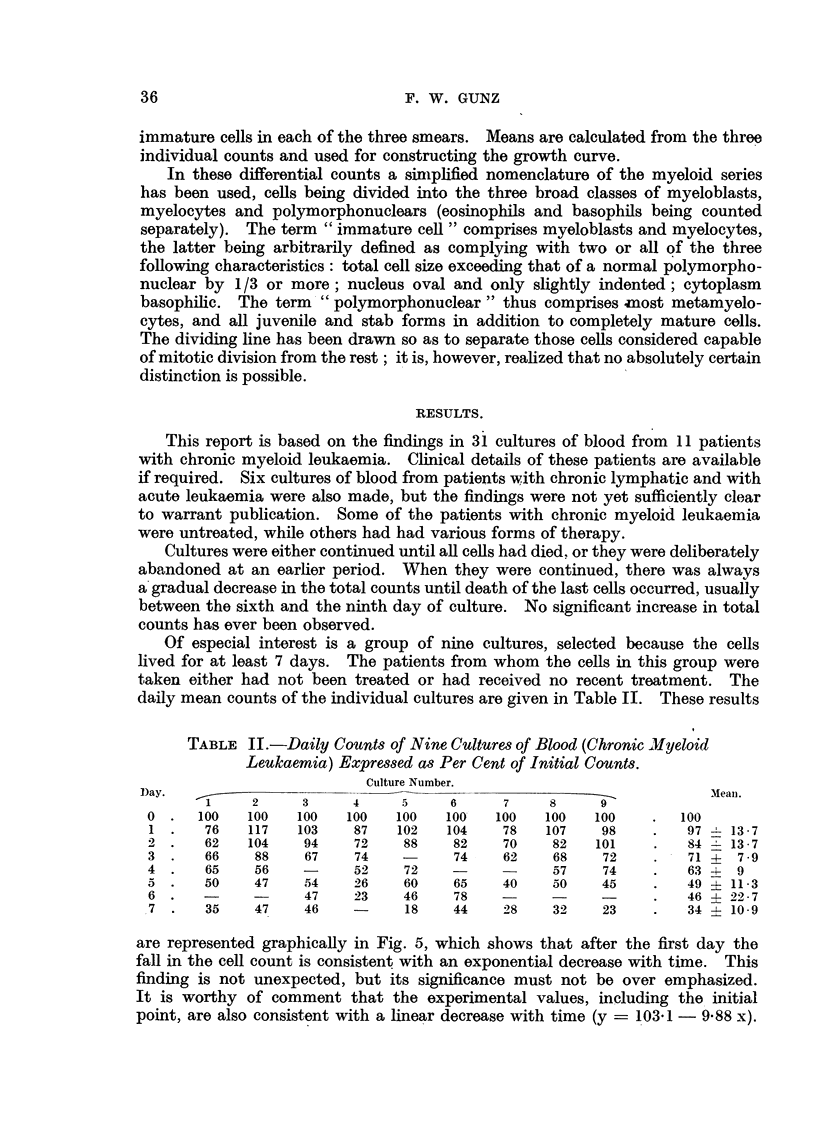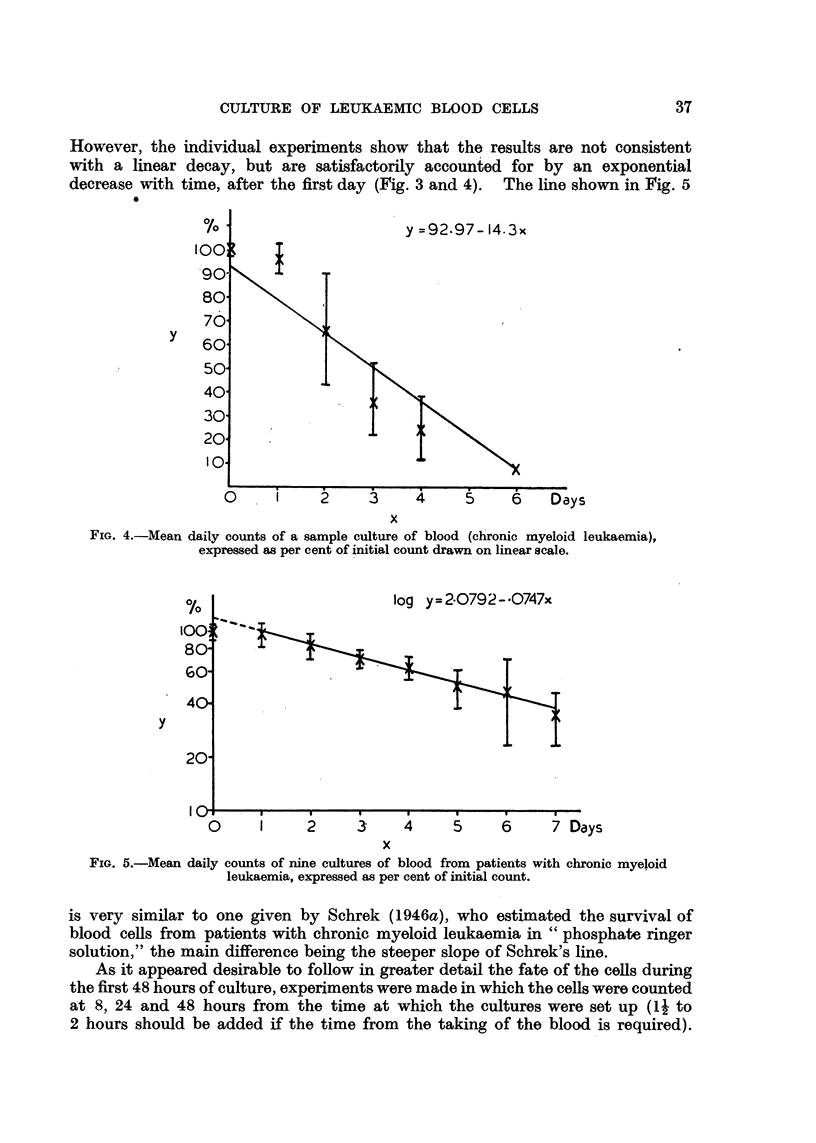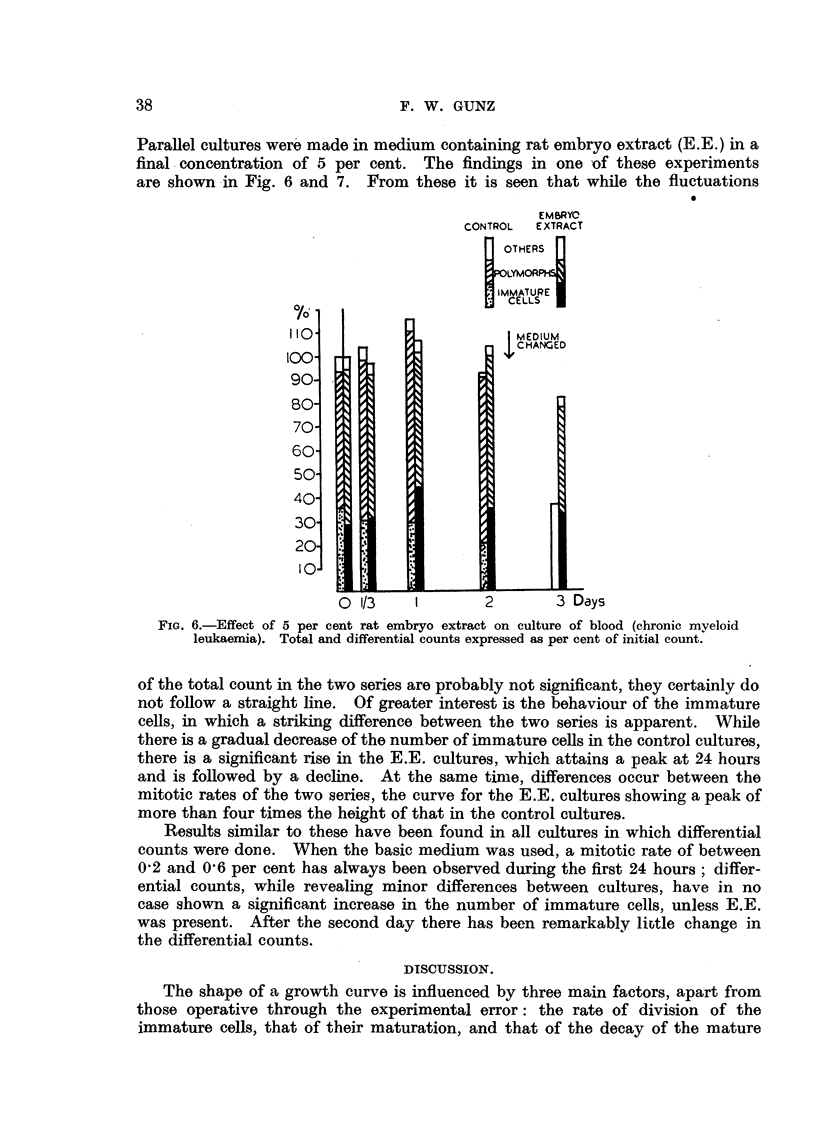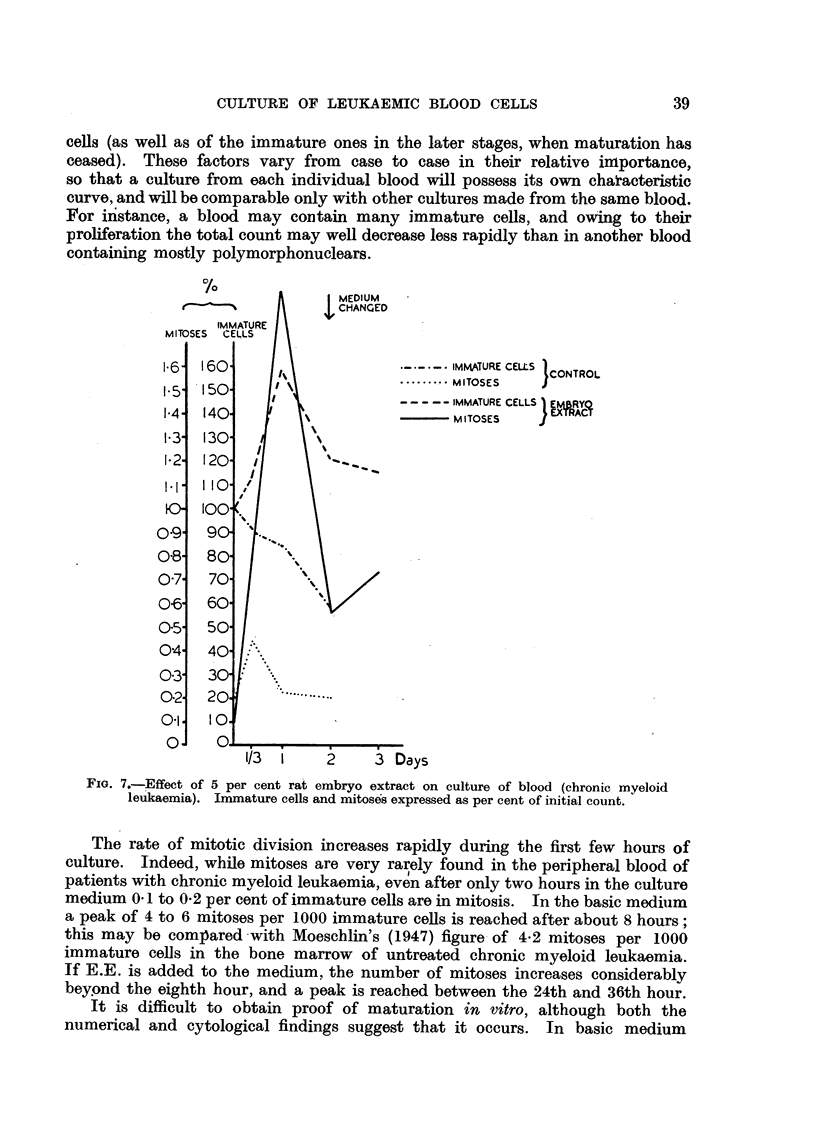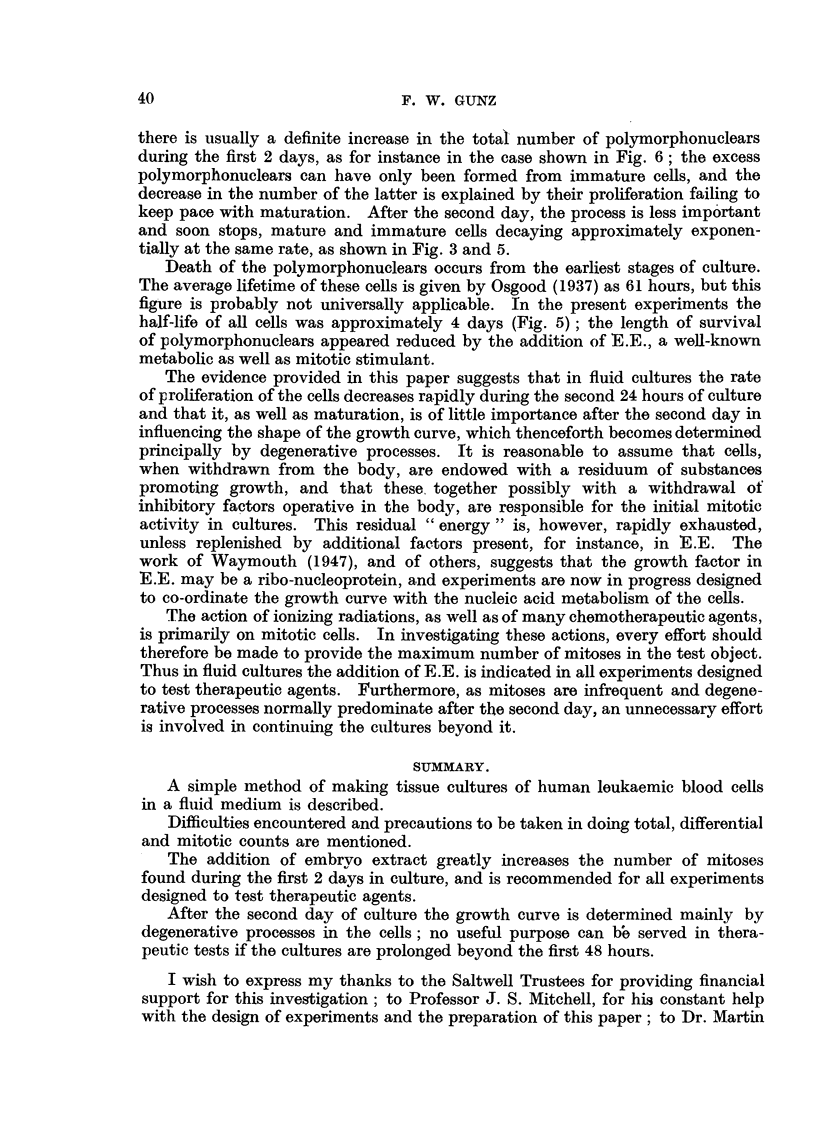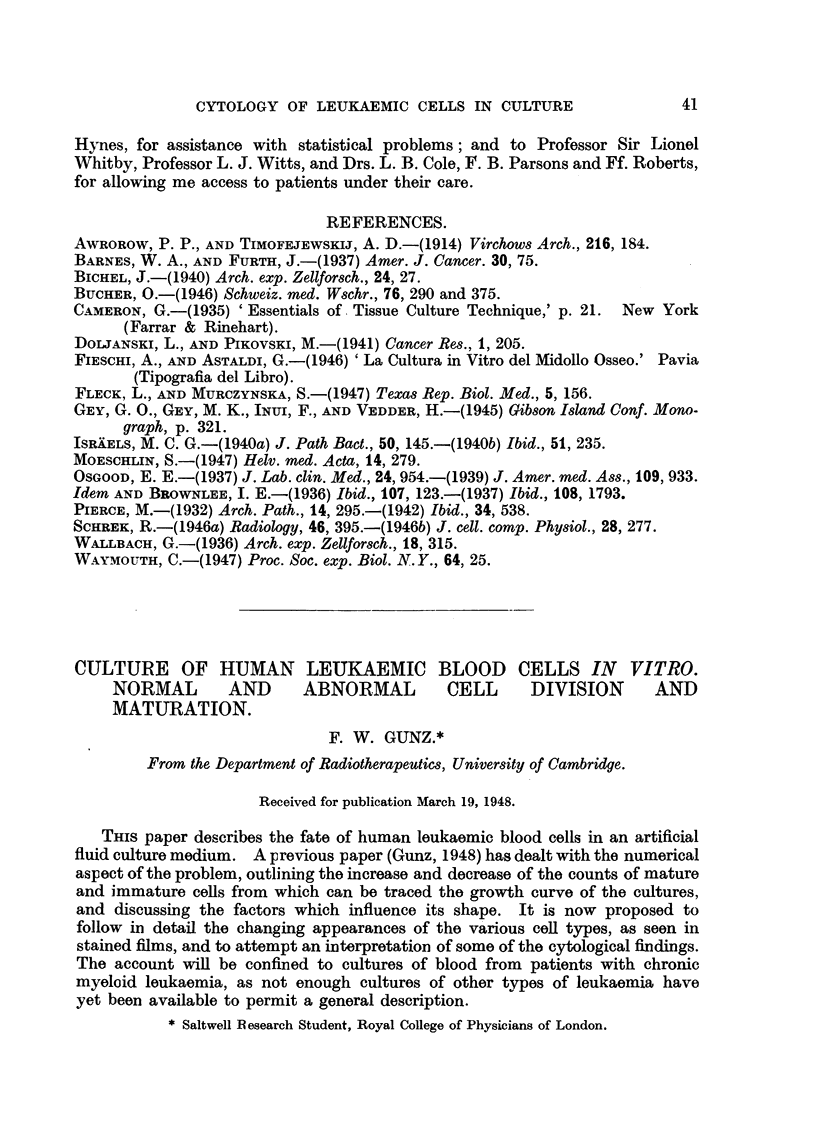# Culture of Human Leukaemic Blood Cells In Vitro; Technique and the Growth Curve

**DOI:** 10.1038/bjc.1948.5

**Published:** 1948-03

**Authors:** F. W. Gunz


### CULTURE OF HUMAN LEUKAEMIC BLOOD CELLS *IN VITRO*; TECHNIQUE AND THE GROWTH CURVE.

F. W. GUNZ.*

*From the Department of Radiotherapeutics, University of Cambridge.*

Received for publication February 28, 1948.

The aim of the work reported in this paper was to perfect a simple and reliable technique for the culture of human leukaemic blood cells, and with it to construct a quantitative base line which would later be available for testing the effects *in vitro* of therapeutic measures such as ionizing radiations or chemical agents. These effects express themselves as changes in the number as well as the appearance of various cell types. Furthermore, the effect on cells in mitosis is of particular interest and importance.

The results which are here reported provide the necessary controls for experiments with therapeutic agents, by establishing the rate of growth and decay

* Saltwell Research Student, Royal College of Physicians of London.

of untreated leukaemic cells observed *in vitro*. Only the quantitative aspects of the subject will be dealt with here ; the cytological findings will be described in a separate paper.

Normal and leukaemic blood and bone marrow have been studied in tissue cultures by many workers since the publication of the first report on tissue culture of leukaemic blood by Awrorow and Timofejewskij (1914). Among other investigations carried out in recent years the following should be mentioned, as they deal wholly or in part with culture of leukaemic blood or marrow : papers by Barnes and Furth (1937) and Bichel (1940) on mouse leukaemia; Doljanski and Pikovski (1941) on fowl leukosis ; and Fieschi and Astaldi (1946), Isräels (1940*a*, 1940*b*), Osgood and Brownlee (1936, 1937, as well as many later papers from the same laboratory), Pierce (1932, 1942), Schrek (1946*a*, 1946*b*), and Wallbach (1936), on human leukaemia. Two main culture methods have been employed : On the one hand, cultivation of solid fragments of marrow or of coagulated buffy coat from the peripheral blood, either in hanging drops or in tubes, which has been favoured by most workers ; on the other, the fluid method originated by Osgood and used later by Isräels and recently by Schrek. A third alternative is cultivation on the chick chorio-allantoic membrane, as suggested by Pierce (1942).

In the investigation of quantitative changes in a given cell population the fluid method offers certain advantages, as it allows of total, as well as of differential cell counts. It is therefore possible to determine the absolute numbers of the various cell types, as well as of mitoses, present at any moment, to note their increase or decrease under experimental conditions, and to observe changes under the influence of therapeutic agents. While much work has been done on these lines on bone-marrow cells, mainly by Osgood and his collaborators, few similar data exist for leukaemic blood. Schrek, one of the few investigators in this field, employed a saline culture medium which did not allow of cell division, so that his valuable findings are not entirely comparable with those obtained by the use of an enriched medium.

### TECHNIQUE.

The technique has been modified from that described by Osgood and Brownlee, the main changes being a simplification of the composition of the culture medium and of the procedure in setting up the cultures. The principle of the method is the distribution of small equal quantities of blood cells, suitably diluted with culture medium, over a series of culture tubes, the whole process being carried out by means of hypodermic syringes.

### *Apparatus.*

The following are required : Screw-cap bottles of the McCartney type, to hold 25–30 ml. ; test-tubes $1\frac{1}{2} \times 7$ cm. ; culture tubes (round-bottomed tubes of hard glass, $2\frac{1}{2} \times 7\frac{1}{2}$ cm., with a neck which can be fitted with vaccine caps) ; glass caps to cover the vaccine cap and fit the shoulder of the culture tube (both the last items can be easily made by any competent glass-blower) ; rubber vaccine caps to fit the culture and small test-tubes ; 50 and 250 ml. conical flasks, with rubber bungs and glass caps to fit their tops ; 1, 2, 10 and 30 ml. all-glass syringes with suitable needles.

All the glassware should be cleaned by the method in general use in tissue-culture laboratories (Cameron, 1935). The vaccine caps are washed, and as rubber is toxic to living cells, they are then coated with a very thin layer of paraffin wax, by dipping them into a saturated solution of paraffin in petrol ether and wiping off excess after withdrawal. The culture tubes are assembled as shown in Fig. 1, a No. 1 hypodermic needle being passed through each vaccine

FIG. 1.—Culture tube ready for introduction of medium.

cap ; the short test-tubes are similarly capped ; they and the screw-cap bottles are fitted with needles and cellophane caps, and all apparatus is sterilized by autoclaving.

*Culture medium.*

This consists of equal parts of Ringer solution at pH 7·3 and of unfiltered reconstituted adult human plasma, as supplied in dried form by the National Blood Transfusion Service. Preliminary experiments showed that there was no significant difference between the results obtained by the use of human placental serum as recommended by Osgood, fresh adult serum and dried and reconstituted plasma. The latter offers the advantage of ready availability in sterile form, and of constant composition. After reconstitution it has been kept for weeks frozen at —20° C., without a detectable change in its properties.

*Setting up of cultures.*

Venous blood is taken under sterile precautions into screw-cap bottles containing enough heparin to give a final concentration of 1:15,000. The quantity of blood required varies between 5 and 30 ml., according to the height of the patient's leucocyte count and the number of tubes to be set- up. The bottle is allowed to stand in the incubator at 37° C. for ½ to 1 hour, when most of the erythrocytes will have settled, the leucocytes and platelets remaining suspended in the supernatant layer. This is now removed and transferrred to a short test-tube, in which it is centrifuged for 1 to 2 minutes at 500 r.p.m. The supernatant plasma is removed, and the cells are washed once in Ringer solution. All the manipulations are carried out by means of syringes, none of the containers being opened in the process. Meanwhile, a suitable quantity of culture medium has been put into a conical flask, and the cells are now added to it, the aim being to give a final concentration of about 2000 leucocytes per c.mm. Some practice is required in judging the correct volume of cells to be added. Three consecutive counts are done on the cell suspension, and when the mean reveals a suitable cell concentration, a quantity of 2 ml. is introduced into each culture tube, care being taken to avoid settling of the cells in the conical flask by shaking the latter at frequent intervals. The No. 1 needles are now removed from the culture tubes, which are incubated at 37° C.

The process of setting up 36 to 48 cultures by this method requires about 1½ hours from the moment of taking the blood, working with an assistant. There is always the danger of contamination of the cultures, although the "closed" method minimizes it, and if working conditions should be unfavourable, it may be necessary to add penicillin to the cultures in a final concentration of 100 units per ml. There is good evidence (Gey, Gey, Inui and Vedder, 1945; Bucher, 1946) that crystalline penicillin has no deleterious influence on tissue cultures. Streptomycin has also been employed, but it may possibly damage the cells.

*Changing of culture medium.*

In the early experiments the culture medium was changed every 2 or 3 days, as recommended by Osgood (1939) and Isräels, but it was found that replacement of the old by an exactly equal quantity of fresh medium was difficult and constituted a source of errors in counting, and that infections were easily introduced in the process. Experiments were then made in which the medium was left unchanged, and no significant difference was noted in the results. This is not unexpected, for in the cultures the volume of the medium exceeds that of the cells by a factor of $10^3$ or more, so that it is difficult to imagine the cells being adversely influenced either by exhaustion of the medium or accumulation of metabolites. On the other hand, results have never been satisfactory when the thickness of the fluid layer exceeded 1 cm., owing, presumably, to the slow diffusion of oxygen to, or of $CO_2$ from the cells.

*Counting.*

After thorough shaking of the tubes, counts are done on unstained cells in undiluted suspension, using Thoma or Neubauer chambers. Difficulties arise in the later days of culture owing to degeneration of some of the cells. Many of these become completely non-refractile and ghost-like, and it is this class of cell which loses practically all power of staining in smears. It will therefore not be counted in differential counts, and a grave source of error will thereby be introduced. For this reason, "ghost cells" are not included in total counts, but only cells showing at least a remnant of refractility.

The parameter count of each tube is calculated by counting all leucocytes in 1 $mm.^2$ of the ruling, multiplying by 10 and rounding off to the nearest 100.

When the volume of fluid in each culture tube is small, as in these experiments, it is unsatisfactory to remove repeated samples for counting, as differences may thereby be introduced in the quantities of fluid remaining in the tubes. It should be more desirable to use a fresh part of the series of tubes for each count, discarding it after counting, provided that it can be shown that the distribution of counts over the total series of tubes is approximately normal, and that the differences arising between individual tubes in the course of the experiment remain reasonably small. The following experiments were designed to test these questions, duplicates being done in each case :

*Experiment* 1.

Blood was obtained and treated as described, until the final leucocyte suspension had been made. A series of 36 counts was then done on it, the same syringe being used for all. The results are shown in Fig. 2, from which it can be seen

FIG. 2.—Frequency distribution of 36 counts of a given W.B.C. suspension. Mean = 2090 ± 208/c.mm.

that the distribution is approximately normal. The large standard deviation of ± 10 per cent should be noted. This is chiefly due to the fact that in the initial stages of the culture there occurs a remarkable clumping of the leucocytes, agglutinates of three to five cells being often observed. This phenomenon, which is variable in degree, has largely disappeared after 24 hours. It is probably analogous to " leukergy " observed in inflammatory states by Fleck and Murczynska (1947).

*Experiment* 2.

A series of 36 cultures was set up as described. The mean of three counts of the final cell suspension, before distribution, was taken as the 100 per cent initial value for all tubes. After 24 hours, six tubes were removed and five counts done on each. Six fresh tubes were similarly counted on each of the next 3 days. No count was done on the fifth day, and a single count on each of the remaining twelve tubes on the sixth day. The standard errors between individual counts and between tubes were calculated for each of the first 4 days. The results are shown in Table I, and graphically in Fig. 3. Table I shows that after the first day the standard error between tubes became somewhat large. This was often due to one or two individual tubes getting " out of step " with the others, probably owing to some special factor operating only in them ; examples are tubes 12 on the second and 24 on the fourth day. The standard error between counts (the experimental error) remained consistently smaller than that between tubes. In spite of the large size of the latter, the trend of the curve is clear from Fig. 3. It is to be noted that results are consistent with an exponential decrease with time of the initial cell count, after the first day of culture.

FIG. 3.—Mean daily counts of a sample culture of blood (chronic myeloid leukaemia), expressed as per cent of initial count drawn on logarithmic scale.

In the light of these two experiments, the following routine procedure has been thought justifiable: The mean of three counts of the original cell suspension is taken as the 100 per cent initial value for all tubes. On each subsequent occasion three separate counts are done on each of three fresh tubes, and the mean and standard error between tubes are calculated for all nine counts. The growth curve is constructed from the resultant points.

*Differential and mitotic counts.*

After the total counts have been done, all the fluid remaining in the culture tubes is transferred to Wintrobe tubes and centrifuged for one minute at 500 r.p.m. The supernatant is removed, and the cells are resuspended in approximately twice their own volume of supernatant and mixed as thoroughly as possible. Smears are then made and stained as desired. Differential counts of 500 cells are done on one smear from each tube (1500 for the three tubes routinely used) ; total and differential counts are also done of all mitoses found in 1000

immature cells in each of the three smears. Means are calculated from the three individual counts and used for constructing the growth curve.

In these differential counts a simplified nomenclature of the myeloid series has been used, cells being divided into the three broad classes of myeloblasts, myelocytes and polymorphonuclears (eosinophils and basophils being counted separately). The term " immature cell " comprises myeloblasts and myelocytes, the latter being arbitrarily defined as complying with two or all of the three following characteristics : total cell size exceeding that of a normal polymorphonuclear by 1/3 or more ; nucleus oval and only slightly indented ; cytoplasm basophilic. The term " polymorphonuclear " thus comprises most metamyelocytes, and all juvenile and stab forms in addition to completely mature cells. The dividing line has been drawn so as to separate those cells considered capable of mitotic division from the rest ; it is, however, realized that no absolutely certain distinction is possible.

### RESULTS.

This report is based on the findings in 31 cultures of blood from 11 patients with chronic myeloid leukaemia. Clinical details of these patients are available if required. Six cultures of blood from patients with chronic lymphatic and with acute leukaemia were also made, but the findings were not yet sufficiently clear to warrant publication. Some of the patients with chronic myeloid leukaemia were untreated, while others had had various forms of therapy.

Cultures were either continued until all cells had died, or they were deliberately abandoned at an earlier period. When they were continued, there was always a gradual decrease in the total counts until death of the last cells occurred, usually between the sixth and the ninth day of culture. No significant increase in total counts has ever been observed.

Of especial interest is a group of nine cultures, selected because the cells lived for at least 7 days. The patients from whom the cells in this group were taken either had not been treated or had received no recent treatment. The daily mean counts of the individual cultures are given in Table II. These results

TABLE II.—*Daily Counts of Nine Cultures of Blood (Chronic Myeloid Leukaemia) Expressed as Per Cent of Initial Counts.*

| Day. | Culture Number. | | | | | | | | | Mean. |
|---|---|---|---|---|---|---|---|---|---|---|
| | 1 | 2 | 3 | 4 | 5 | 6 | 7 | 8 | 9 | |
| 0 | 100 | 100 | 100 | 100 | 100 | 100 | 100 | 100 | 100 | 100 |
| 1 | 76 | 117 | 103 | 87 | 102 | 104 | 78 | 107 | 98 | 97 ± 13·7 |
| 2 | 62 | 104 | 94 | 72 | 88 | 82 | 70 | 82 | 101 | 84 ± 13·7 |
| 3 | 66 | 88 | 67 | 74 | — | 74 | 62 | 68 | 72 | 71 ± 7·9 |
| 4 | 65 | 56 | — | 52 | 72 | — | — | 57 | 74 | 63 ± 9 |
| 5 | 50 | 47 | 54 | 26 | 60 | 65 | 40 | 50 | 45 | 49 ± 11·3 |
| 6 | — | — | 47 | 23 | 46 | 78 | — | — | — | 46 ± 22·7 |
| 7 | 35 | 47 | 46 | — | 18 | 44 | 28 | 32 | 23 | 34 ± 10·9 |

are represented graphically in Fig. 5, which shows that after the first day the fall in the cell count is consistent with an exponential decrease with time. This finding is not unexpected, but its significance must not be over emphasized. It is worthy of comment that the experimental values, including the initial point, are also consistent with a linear decrease with time ($y = 103{\cdot}1 - 9{\cdot}88\ x$).

However, the individual experiments show that the results are not consistent with a linear decay, but are satisfactorily accounted for by an exponential decrease with time, after the first day (Fig. 3 and 4). The line shown in Fig. 5

Fig. 4.—Mean daily counts of a sample culture of blood (chronic myeloid leukaemia), expressed as per cent of initial count drawn on linear scale.

Fig. 5.—Mean daily counts of nine cultures of blood from patients with chronic myeloid leukaemia, expressed as per cent of initial count.

is very similar to one given by Schrek (1946*a*), who estimated the survival of blood cells from patients with chronic myeloid leukaemia in " phosphate ringer solution," the main difference being the steeper slope of Schrek's line.

As it appeared desirable to follow in greater detail the fate of the cells during the first 48 hours of culture, experiments were made in which the cells were counted at 8, 24 and 48 hours from the time at which the cultures were set up ($1\frac{1}{2}$ to 2 hours should be added if the time from the taking of the blood is required).

Parallel cultures were made in medium containing rat embryo extract (E.E.) in a final concentration of 5 per cent. The findings in one of these experiments are shown in Fig. 6 and 7. From these it is seen that while the fluctuations

FIG. 6.—Effect of 5 per cent rat embryo extract on culture of blood (chronic myeloid leukaemia). Total and differential counts expressed as per cent of initial count.

of the total count in the two series are probably not significant, they certainly do not follow a straight line. Of greater interest is the behaviour of the immature cells, in which a striking difference between the two series is apparent. While there is a gradual decrease of the number of immature cells in the control cultures, there is a significant rise in the E.E. cultures, which attains a peak at 24 hours and is followed by a decline. At the same time, differences occur between the mitotic rates of the two series, the curve for the E.E. cultures showing a peak of more than four times the height of that in the control cultures.

Results similar to these have been found in all cultures in which differential counts were done. When the basic medium was used, a mitotic rate of between 0·2 and 0·6 per cent has always been observed during the first 24 hours ; differential counts, while revealing minor differences between cultures, have in no case shown a significant increase in the number of immature cells, unless E.E. was present. After the second day there has been remarkably little change in the differential counts.

### DISCUSSION.

The shape of a growth curve is influenced by three main factors, apart from those operative through the experimental error : the rate of division of the immature cells, that of their maturation, and that of the decay of the mature cells (as well as of the immature ones in the later stages, when maturation has ceased). These factors vary from case to case in their relative importance, so that a culture from each individual blood will possess its own characteristic curve, and will be comparable only with other cultures made from the same blood. For instance, a blood may contain many immature cells, and owing to their proliferation the total count may well decrease less rapidly than in another blood containing mostly polymorphonuclears.

FIG. 7.—Effect of 5 per cent rat embryo extract on culture of blood (chronic myeloid leukaemia). Immature cells and mitoses expressed as per cent of initial count.

The rate of mitotic division increases rapidly during the first few hours of culture. Indeed, while mitoses are very rarely found in the peripheral blood of patients with chronic myeloid leukaemia, even after only two hours in the culture medium 0·1 to 0·2 per cent of immature cells are in mitosis. In the basic medium a peak of 4 to 6 mitoses per 1000 immature cells is reached after about 8 hours; this may be compared with Moeschlin's (1947) figure of 4·2 mitoses per 1000 immature cells in the bone marrow of untreated chronic myeloid leukaemia. If E.E. is added to the medium, the number of mitoses increases considerably beyond the eighth hour, and a peak is reached between the 24th and 36th hour.

It is difficult to obtain proof of maturation *in vitro*, although both the numerical and cytological findings suggest that it occurs. In basic medium there is usually a definite increase in the total number of polymorphonuclears during the first 2 days, as for instance in the case shown in Fig. 6 ; the excess polymorphonuclears can have only been formed from immature cells, and the decrease in the number of the latter is explained by their proliferation failing to keep pace with maturation. After the second day, the process is less important and soon stops, mature and immature cells decaying approximately exponentially at the same rate, as shown in Fig. 3 and 5.

Death of the polymorphonuclears occurs from the earliest stages of culture. The average lifetime of these cells is given by Osgood (1937) as 61 hours, but this figure is probably not universally applicable. In the present experiments the half-life of all cells was approximately 4 days (Fig. 5) ; the length of survival of polymorphonuclears appeared reduced by the addition of E.E., a well-known metabolic as well as mitotic stimulant.

The evidence provided in this paper suggests that in fluid cultures the rate of proliferation of the cells decreases rapidly during the second 24 hours of culture and that it, as well as maturation, is of little importance after the second day in influencing the shape of the growth curve, which thenceforth becomes determined principally by degenerative processes. It is reasonable to assume that cells, when withdrawn from the body, are endowed with a residuum of substances promoting growth, and that these together possibly with a withdrawal of inhibitory factors operative in the body, are responsible for the initial mitotic activity in cultures. This residual " energy " is, however, rapidly exhausted, unless replenished by additional factors present, for instance, in E.E. The work of Waymouth (1947), and of others, suggests that the growth factor in E.E. may be a ribo-nucleoprotein, and experiments are now in progress designed to co-ordinate the growth curve with the nucleic acid metabolism of the cells.

The action of ionizing radiations, as well as of many chemotherapeutic agents, is primarily on mitotic cells. In investigating these actions, every effort should therefore be made to provide the maximum number of mitoses in the test object. Thus in fluid cultures the addition of E.E. is indicated in all experiments designed to test therapeutic agents. Furthermore, as mitoses are infrequent and degenerative processes normally predominate after the second day, an unnecessary effort is involved in continuing the cultures beyond it.

### SUMMARY.

A simple method of making tissue cultures of human leukaemic blood cells in a fluid medium is described.

Difficulties encountered and precautions to be taken in doing total, differential and mitotic counts are mentioned.

The addition of embryo extract greatly increases the number of mitoses found during the first 2 days in culture, and is recommended for all experiments designed to test therapeutic agents.

After the second day of culture the growth curve is determined mainly by degenerative processes in the cells ; no useful purpose can be served in therapeutic tests if the cultures are prolonged beyond the first 48 hours.

I wish to express my thanks to the Saltwell Trustees for providing financial support for this investigation ; to Professor J. S. Mitchell, for his constant help with the design of experiments and the preparation of this paper ; to Dr. Martin

Hynes, for assistance with statistical problems; and to Professor Sir Lionel Whitby, Professor L. J. Witts, and Drs. L. B. Cole, F. B. Parsons and Ff. Roberts, for allowing me access to patients under their care.

### REFERENCES.

AWROROW, P. P., AND TIMOFEJEWSKIJ, A. D.—(1914) *Virchows Arch.*, **216**, 184.
BARNES, W. A., AND FURTH, J.—(1937) *Amer. J. Cancer.* **30**, 75.
BICHEL, J.—(1940) *Arch. exp. Zellforsch.*, **24**, 27.
BUCHER, O.—(1946) *Schweiz. med. Wschr.*, **76**, 290 and 375.
CAMERON, G.—(1935) 'Essentials of Tissue Culture Technique,' p. 21. New York (Farrar & Rinehart).
DOLJANSKI, L., AND PIKOVSKI, M.—(1941) *Cancer Res.*, **1**, 205.
FIESCHI, A., AND ASTALDI, G.—(1946) 'La Cultura in Vitro del Midollo Osseo.' Pavia (Tipografia del Libro).
FLECK, L., AND MURCZYNSKA, S.—(1947) *Texas Rep. Biol. Med.*, **5**, 156.
GEY, G. O., GEY, M. K., INUI, F., AND VEDDER, H.—(1945) *Gibson Island Conf. Monograph*, p. 321.
ISRÄELS, M. C. G.—(1940*a*) *J. Path Bact.*, **50**, 145.—(1940*b*) *Ibid.*, **51**, 235.
MOESCHLIN, S.—(1947) *Helv. med. Acta*, **14**, 279.
OSGOOD, E. E.—(1937) *J. Lab. clin. Med.*, **24**, 954.—(1939) *J. Amer. med. Ass.*, **109**, 933.
*Idem* AND BROWNLEE, I. E.—(1936) *Ibid.*, **107**, 123.—(1937) *Ibid.*, **108**, 1793.
PIERCE, M.—(1932) *Arch. Path.*, **14**, 295.—(1942) *Ibid.*, **34**, 538.
SCHREK, R.—(1946*a*) *Radiology*, **46**, 395.—(1946*b*) *J. cell. comp. Physiol.*, **28**, 277.
WALLBACH, G.—(1936) *Arch. exp. Zellforsch.*, **18**, 315.
WAYMOUTH, C.—(1947) *Proc. Soc. exp. Biol. N.Y.*, **64**, 25.